# INTEGRATED BEHAVIORAL AND PSYCHOLOGICAL INTERVENTIONS IN VHA COMMUNITY LIVING CENTERS

**DOI:** 10.1093/geroni/igae098.3434

**Published:** 2024-12-31

**Authors:** Kyle Page, HyeRim Ryu, Elizabeth MacDonald, Benjamin Szymanski, Kimberly Curyto, Michele Karel

**Affiliations:** Edward Hines, Jr. VA Hospital, Oak Park, Illinois, United States; Edward Hines, Jr. VA Hospital, Oak Park, Illinois, United States; Office of Mental Health and Suicide Prevention, Department of Veteran Affairs, Ann Arbor, Michigan, United States; Office of Mental Health and Suicide Prevention, Department of Veterans Affairs, Ann Arbor, Michigan, United States; Western New York VA Healthcare System, Batavia, New York, United States; VA Central Office, Washington, District of Columbia, United States

## Abstract

Veterans Health Administration (VHA) Community Living Centers (CLC), or nursing home care settings, care for Veterans with high rates of mental illness and behavioral concerns. VHA requires the integration of psychologists on CLC teams and access to psychiatric services. An anonymous national survey of CLC mental health professionals aimed to characterize the status of CLC mental health practice, including intervention services. One hundred seven of 221 (48.4%) psychologists and psychiatric providers responded to the survey. Half or more of respondents reported these psychological interventions to be most helpful in the CLC: supportive therapy (76%), behavior therapy (75%), behavior management (66%), reminiscence/narrative therapy (65%), relaxation training (59%), cognitive behavioral therapy (54%), problem-solving therapy (52%), and motivational interviewing (50%). Respondents also identified barriers to utilizing Evidence Based Psychotherapies (EBPs) in the CLC, including Veteran cognitive impairment (57%), illness (48%), and preference for another form of intervention (32%). About a third (32%) reported offering group interventions. Respondents reported frequent collaboration with CLC team members in mental health interventions, most commonly by asking about their observations (76%), co-signing others on progress notes (71%), having them monitor signs and symptoms (68%), and many other strategies for team-based care. One third reported having regular, interdisciplinary behavior rounds and 67% reported that their teams used a team-based behavioral intervention (“STAR-VA”) for managing dementia-related behaviors. Findings suggest a wide range of mental health intervention practice patterns in the CLC, the importance of team engagement, and opportunities for additional clinical and training resources.

